# Cerebrospinal Fluid Biomarkers in Alzheimer’s Disease—From Brain Starch to Bench and Bedside

**DOI:** 10.3390/diagnostics7030042

**Published:** 2017-07-13

**Authors:** Matthias Pawlowski, Sven G. Meuth, Thomas Duning

**Affiliations:** Department of Neurology, University Hospital Münster, Albert-Schweitzer-Campus 1, Building A1, Münster 48149, Germany

**Keywords:** Alzheimer’s disease, dementia, fluid biomarkers, cerebrospinal fluid, amyloid-β, tau

## Abstract

Alzheimer’s disease is the most common cause of dementia. Over the last three decades, research has advanced dramatically and provided a detailed understanding of the molecular events underlying the pathogenesis of Alzheimer’s disease. In parallel, assays for the detection of biomarkers that reflect the typical Alzheimer’s disease-associated pathology have been developed and validated in myriads of clinical studies. Such biomarkers complement clinical diagnosis and improve diagnostic accuracy. The use of biomarkers will become even more important with the advent of disease-modifying therapies. Such therapies will likely be most beneficial when administered early in the disease course. Here, we summarise the development of the core Alzheimer’s disease cerebrospinal fluid biomarkers: amyloid-β and tau. We provide an overview of their role in cellular physiology and Alzheimer’s disease pathology, and embed their development as cerebrospinal fluid biomarkers into the historical context of Alzheimer’s disease research. Finally, we summarise recommendations for their use in clinical practice, and outline perspectives for novel cerebrospinal fluid candidate biomarkers.

## 1. Introduction

Alzheimer’s disease (AD) is the most common cause of dementia [1]. It is a slowly progressive neurodegenerative disorder with an insidious onset marked by prominent initial impairment of episodic memory [2]. The disease course is characterised by progressive deterioration of episodic memory and the successive impairment of additional cognitive domains, including semantic memory, executive dysfunction, apraxia, visuospatial and visuoperceptive deficits, thus giving rise to the full dementia syndrome [3]. The number of patients suffering from AD is expected to increase dramatically over the next decades because of the aging population [1]. Thus, the socioeconomic burden of AD, and the demand for discovering appropriate treatment options will continue to grow [1].

Neurodegeneration in AD is estimated to start 20–30 years before the onset of clinical symptoms [4,5,6,7]. The characteristic neuropathological changes that are found in patients with AD are the abundant senile plaques, composed of amyloid-β (Aβ), and neurofibrillary tangles (NFTs), neuropil threads, and dystrophic neurites composed of hyperphosphorylated tau [6]. Astrogliosis and microglial cell activation are typically associated with plaques and tangles [6]. Other characteristic neuropathological features include congophilic amyloid angiopathy and certain patterns of neuronal and synaptic loss [6]. Today, many pathogenic cascades leading to Aβ generation and aggregation have been unveiled, and mechanisms underlying erroneous tau protein homeostasis have been deciphered [8]. These scientific advances have enabled the development of novel treatment strategies with disease-modifying potential. Today, many drug candidates targeting different steps in the pathophysiological cascade of plaque or tangle formation are in clinical treatment trials. Treatment strategies targeting Aβ currently focus on the inhibition of cleavage of the amyloid precursor protein (APP), thus reducing Aβ production (secretase inhibitors), Aβ aggregation inhibitors, immunisation with Aβ, and the passive immunisation with Aβ-antibodies [9]. Strategies for the reduction of misfolded tau-mediated AD neuropathology are directed to correct a loss of tau function resulting from sequestration of cellular tau and minimize possible gain-of-function toxicities caused by multimeric tau species [10]. It is conceivable that these drugs are probably most effective in the earlier stages of the disease, when plaque and tangle load and neurodegeneration are less advanced [11]. Moreover, treatments labelled as “disease-modifiers” must show a beneficial effect on clinical parameters and must also affect the central disease pathology [11,12]. These challenges have initiated a concerted search of the research community for biomarkers that adequately mirror core elements of the disease process, serve as diagnostic tools, especially for early diagnosis, and identify and monitor the biochemical effects of the respective drug candidate [11,13].

Here, we summarise the development and current role of cerebrospinal fluid (CSF) biomarkers for the diagnosis of AD. We place the development of the established core biomarkers in the CSF, namely Aβ and tau, into the historical context of their discovery, and summarise their current role in clinical practice. Finally, we discuss additional and novel CSF biomarkers reflecting pathological disease processes in AD.

## 2. Amyloid-β

Aβ is a physiological product of cellular metabolism. It is generated through successive cleavage steps of the amyloid precursor protein (APP) by β- and γ-secretases [14]. Because of the central role of APP and its derivatives, including Aβ, in AD pathogenesis, major research efforts have been invested to understand its physiological and pathological functions. In vitro and in vivo studies have shed light on the manifold cellular functions of APP, which can be executed either by the full-length APP protein, or as one of its cleavage derivatives [15].

### 2.1. APP and Aβ—Biochemistry and Physiology

**Figure 1:**
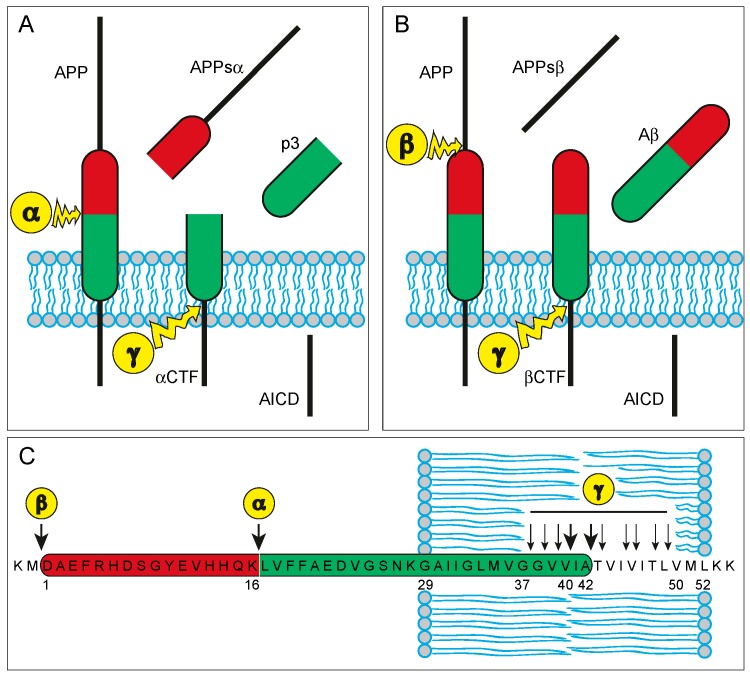
Proteolytic processing of the amyloid precursor protein. (**A**) The non-amyloidogenic pathway: sequential cleavage of amyloid precursor protein (APP) by α- and γ-secretases yields the soluble APP ectodomain α (APPsα), the 3 kDA peptide (p3), and the APP intracellular domain (AICD); (**B**) The amyloidogenic pathway: sequential cleavage of APP by β- and γ-secretases yields the soluble APP ectodomain β (APPsβ), amyloid β (Aβ), and AICD; (**C**) Cleavage sites within the Aβ-region of APP: In the non-amyloidogenic pathway, APP is first cut between residues Lys_16_ and Leu_17_ within the amyloid-β region of APP by α-secretase activity. In the amyloidogenic pathway, APP is first cut by β-secretase at the amino-terminus of Aβ. In both pathways, the second cleavage is catalysed by γ-secretase activity within the transmembrane domain of APP. In contrast to APP processing by α- and β-secretase, γ-secretase activity is not restricted to a single site. Rather, γ-secretase cleaves three times successively within the transmembrane domain at the ε-, z-, and γ-sites. Different ε-, ζ-, and γ-sites exist (ε-sites: Aβ_48_, Aβ_49_; ζ-sites: Aβ_45_, Aβ_46_; γ-sites: Aβ_37_, Aβ_38_, Aβ_39_, Aβ_40_, Aβ_42_, Aβ_43_). Depending on the actual γ-site, different carboxy-terminal truncated isoforms of Aβ are generated (under physiological conditions: Aβ_40_ 90%; Aβ_42_ < 10%; Aβ_37_ and Aβ_38_ minor amounts). In AD, this approximated ratio is shifted towards Aβ_42_, which is more prone to aggregation and represents the predominant isoform in amyloid plaques. Abbreviations: APP = amyloid precursor protein; APPsα/β = APP soluble ectodomains; α/βCTF = carboxy-terminal fragments; p3 = 3 kDa peptide; AICD = APP intracellular domain; α = α-secretase; β = β-secretase; γ = γ-secretase [14].

APP is an integral type I transmembrane protein with a single transmembrane domain, a large extracellular ectodomain, and a short cytoplasmic tail (Figure 1). In humans, APP is encoded by the *APP* (amyloid β precursor protein) gene on the long arm of chromosome 21 (21q21.3). APP is ubiquitously expressed, with particularly high expression levels in neurons [16]. The protein is located within neuronal cell bodies, in dendrites, and in axons. Within axons, it undergoes rapid anterograde transport, and is targeted in vesicles to synaptic sites [15,17,18,19].

APP undergoes enzymatic cleavage, which is initiated either by α-secretase within the Aβ region of APP, or through β-secretase (BACE) at the amino-terminus of the Aβ region, leading to the secretion of large soluble ectodomains termed APPsα and APPsβ, respectively (Figure 1) [14]. The remaining carboxy-terminal fragments (CTFα and CTFβ, respectively) are cleaved in a subsequent step through the enzymatic activity of γ-secretase and result in the production of either Aβ (from CTFβ) or p3 (from CTFα), and—in both cases—the APP intracellular domain (AICD; Figure 1) [14]. The γ-secretase cleavage sites reside within the transmembrane domain of APP [14]. In contrast to the α- and β-secretases, γ-secretase does not cleave a single peptide bond, but instead, it cleaves three successive times within the transmembrane domain at the so called ε-, ζ-, and γ-sites [14]. The last of the three γ-secretase cuts occurs at one of several possible γ-cleavage sites (Aβ_37_, Aβ_38_, Aβ_39_, Aβ_40_, Aβ_42_, or Aβ_43_), which, in comparison to ε- and ζ-sites, are closest towards the Aβ amino-terminus, thus creating different carboxy-terminal truncated isoforms of Aβ (Figure 1) [14]. Under physiological conditions, neurons generate mainly the shorter isoform ending at the valine residue 40 (Aβ_40_), and the slightly longer form ending at alanine 42 (Aβ_42_), whereas other isoforms such as Aβ_37_ and Aβ_38_ are produced only in minor amounts [14]. The production of Aβ is normally counterbalanced by its elimination, which occurs mainly through proteolytic degradation, but also via cell-mediated clearance, passive and active transport, and the aggregation and deposition of Aβ into insoluble aggregates [20].

APP exerts cellular and synaptic adhesion functions. The extracellular domain of APP interacts with a variety of extracellular matrix components, including heparin [21], collagen type I [22], and laminin [23], indicating a role of APP in cell-matrix adhesion [15]. Moreover, APP can promote cell-cell adhesion through the formation of transcellular antiparallel APP/APP dimers [24]. Like other synaptic adhesion molecules, the extracellular sequence of APP mediates trans synaptic interaction, whereas the intracellular domain is responsible for the recruitment of pre- or postsynaptic complexes to the nerve terminals [25]. In addition to its direct role in cellular and synaptic adhesion, APP co-localises with other cell surface adhesion molecules, such as integrins [26], and neural cell adhesion molecule (NCAM) [27], potentially modulating their adhesive function [15]. Largely mediated by its adhesion properties, APP exerts trophic support for neurons and synapses [15]. Indeed, its deletion or reduction is associated with impaired neurite outgrowth, decreased neuronal viability, and reduced synaptic activity [28,29]. The growth promoting, synaptotrophic and neuroprotective functions may be exerted, in part, both by the full-length APP protein and by its α-secretase cleaved soluble fragment APPsα [30]. In contrast, the slightly shorter β-secretase cleavage product (APPsβ) is considerably less active and may even be neurotoxic [31]. Finally, γ-secretase cleavage not only yields the extracellular products Aβ and p3 (derived from βCTF and αCTF, respectively), but also releases the AICD, which was proposed to participate in the formation of larger complexes that translocate into the nucleus to act as transcriptional activators and histone-modifiers [15,32,33].

### 2.2. APP and Aβ—Their Role in the Neuropathology of AD

Based on the identification of Aβ as the core protein component of senile plaques and mutations in the *APP* gene in some familial cases of early onset AD, Aβ has been proposed as the driving force in the pathogenesis of AD. This idea was summarised in the “amyloid cascade hypothesis” [34,35,36]. It is believed that an imbalance between the production and clearance of Aβ is the initiating event in AD pathogenesis [20]. Excessive amounts of soluble and hydrophobic Aβ assemblies bind directly to different components of neuronal and non-neuronal plasma membranes within the CNS, triggering multiple molecular mediators affecting diverse intracellular pathways that ultimately orchestrate synaptic dysfunction and neuronal degeneration [37].

### 2.3. From Brain Starch to the Use of Aβ as Biomarker for Alzheimer’s Disease—Historical Context

Amyloid is a substance consisting of insoluble fibrils of fibrous protein. It appears homogeneous and amorphous in light microscopy and stains pink with haematoxylin-eosin and metachromatically with methyl or crystal violet. Amyloid reacts with Congo red dye, yielding an apple-green birefringence under polarised light. In electron microscopy, amyloid appears as rigid, linear, non-branching, aggregated fibrils that are 7.5–10 nm in width [38].

The scientific term “amyloid” was coined in 1838 by Matthias Schleiden, a German botanist, who used it to describe a normal, starch-like constituent of plants [39]. Rudolf Virchow first introduced the term “amyloid” into medical literature in 1854. He used it to describe deposits in the central nervous system that exhibited the same staining characteristics as starch following reaction with iodine and sulfuric acid [40]. Virchow concluded that those cerebral structures were made of starch and referred to them as “corpora amylacea” (derived from the Latin and Greek words for starch: “amylum” and “αμυλον”, respectively) [39,41]. The concept of amyloid has transformed several times over the course of the following centuries. Today, it has become clear that the cerebral corpora amylacea reported by Virchow are indeed mostly composed of polyglycosan. However, they do not represent amyloid, in the sense of how amyloid is defined today, and they have nothing in common with the senile plaques that are found in the brains of patients suffering from AD. Still, the term “amyloid” has endured its journey along the labyrinthine paths of amyloid history throughout the centuries and remains in use to denote deposits of fibrillar proteins responsible for damage or functional compromise of different tissues in a variety of human diseases [38].

In 1906, Alois Alzheimer reported the brain autopsy results of his patient Auguste Deter, a woman who had died, aged 55 years, from a previously unrecognised disorder with progressive loss of short-term memory and behavioural abnormalities [42]. Alzheimer noted the presence of two distinctive pathologies: “miliary bodies” (plaques) and “dense bundles of fibrils” (tangles) which we know today, represent the pathological hallmarks of AD [42]. The first descriptions of AD pathology, including those of Alois Alzheimer, were based on Nissl’s silver staining method without any idea about its biochemical composition or a relationship to amyloid of such structures [39]. Indeed, the amyloid nature of senile plaques in AD was described only two decades later [39,43], shortly after the discovery of the Congo red staining for amyloid [44], and the establishment of Congo red reactivity as research criterion for amyloid [45].

Using electron microscopy, Cohen and Calkins reported on fibrillary structures in the samples of several types of amyloids [46]. The subsequent development of methods for the isolation of amyloid fibrils from various tissues [47,48,49] enabled the identification of their β-pleated sheet configuration [50,51], and the identity of their protein core, starting with the identification of immunoglobulin light chains underlying amyloid plaques in “primary” (today: AL) amyloidosis [52]. During the subsequent years, many different amyloid proteins were identified one after another [53]. Glenner and Wong were the first to identify the major protein component of vascular amyloid, a 4 kDa polypeptide now referred to as amyloid β protein (Aβ), which they had derived from meningeal microvessels that were stripped from the brains of AD patients and patients with Down syndrome [54,55]. Subsequent studies established that the same protein was the major component of the cerebral amyloid plaques [56]. The availability of the amino acid sequence of Aβ led to the identification of its precursor protein, the amyloid-β precursor protein, the cloning and sequencing of the amyloid precursor protein (*APP*) gene, and the mapping of its genomic locus on the long arm of chromosome 21 [57,58,59]. Only a few years later, linkage studies unveiled the first defined AD-causing point mutations within the *APP* gene in some pedigrees with early onset autosomal dominant AD [60]. Together, these findings resulted in a series of influential reviews in which the idea of the “Amyloid Cascade Hypothesis” for the pathogenesis of AD was born [34,35,36].

Glenner himself had assumed that there would be a soluble, circulating peptide that served as the subunit of the highly insoluble amyloid fibrils that he first isolated [54,55], thereby stimulating an intensive search for a soluble form of Aβ in biological fluids, including plasma and CSF [61]. The first evidence of a soluble form of Aβ stems from experiments, which revealed the presence of a small (4 kDa), entirely soluble peptide (subsequently identified as Aβ through radiosequencing), in the conditioned medium of human embryonic kidney cells that were stably transfected with APP [62]. Moreover, a slightly smaller, 3 kDa peptide (subsequently designated “p3”) was detected in the same conditioned medium [62]. A following series of experiments showed that a variety of cell types that express APP naturally secrete both Aβ and p3 into the medium under normal metabolic conditions, including human neurons [62]. In parallel, novel and highly sensitive antibodies allowed the first detection of Aβ by enzyme-linked immunosorbant assays (ELISA) in biological fluids, including CSF and plasma [63].

After it was established that Aβ is generated as a soluble protein during normal cellular metabolism and is secreted into the CSF, a series of clinical studies examining CSF Aβ as a candidate biomarker for AD were published [64]. However, these initial reports on Aβ in CSF were based on ELISAs measuring total Aβ levels that did not discriminate between different Aβ isoforms [64]. While some studies found a slight decrease in the CSF level of total Aβ in AD patients [65,66,67], others found no differences when comparing AD patients and healthy controls [68,69,70]. As discussed above, there are several amino-terminally and carboxy-terminally truncated forms of Aβ (Figure 1) [14]. The two major C-terminal variants of Aβ, Aβ_40_ and Aβ_42_, are the result of different cleavage sites of the γ-secretase complex (see above). The longer isoform Aβ_42_ was found to be more prone to aggregation, representing the predominating form of Aβ in senile plaques. These findings led to the focus on immunoassays specifically designed for the detection of the Aβ_42_ isoform in the CSF [64].

### 2.4. Aβ as CSF Biomarker for Alzheimer’s Disease

Several different methods have been developed for quantification of Aβ_42_ in CSF. In patients with AD, a decrease to about 50% of control levels has been found using most of these methods [11]. An increase in CSF Aβ_42_ in AD was found in only one study [71], and is generally attributed to methodological problems, such as assay specificity for aggregated or truncated Aβ variants, or differences in patient and control groups [72]. The reduced CSF levels of Aβ_42_ in AD are hypothesised to be caused by its aggregation and sequestration in cerebral plaques, with less Aβ being available to diffuse into the CSF [11,64]. This is supported by findings from (1) autopsy studies showing that levels of Aβ_42_ in postmortem ventricular CSF negatively correlate with plaque load at autopsy [73]; (2) analysis of antemortem lumbar CSF, with low levels correlating with postmortem plaque load [74]; and (3) functional imaging studies in which cerebral Aβ load is directly visualised by positron emission tomography (PET) using Aβ ligands, such as the 11c-labelled Pittsburgh Compound B (^11c^PIB), with higher Aβ ligand binding correlating with lower CSF Aβ_42_ levels [11,64,75,76,77,78].

## 3. Tau

Tau is a heat stable, hydrophilic, microtubule-associated protein that is primarily located in neuronal axons. Through the binding of tubulin in the axonal microtubules, tau promotes microtubule assembly and stability, exerting important roles for axonal transport and function [79].

### 3.1. Tau—Biochemistry and Physiology

**Figure 2:**
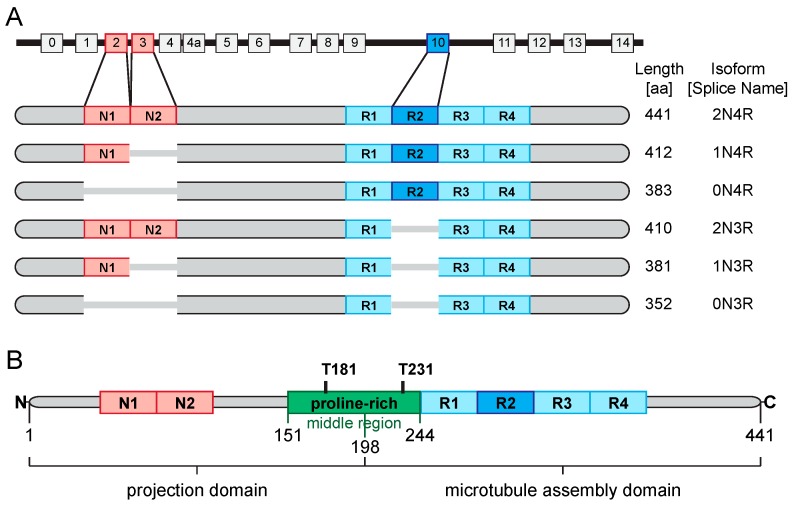
*MAPT* structure, tau isoforms, and tau domains. (**A**) The schematic depicts the structure of the *MAPT* gene containing 16 exons, and the six major tau isoforms that are expressed in the human CNS. Their differences result from alternative splicing of exon 2 (E2), E3 and E10. In the adult human CNS, the concentration of the 3R and 4R isoforms are approximately equal, whereas 0N, 1N and 2N isoforms make up ~37%, ~54% and ~9%, respectively; (**B**) The schematic demonstrates the domain structure of 2N4R tau, which is broadly divided into two major domains: the amino N-terminal projection domain and the carboxy; C-terminal microtubule assembly domain. The latter comprises the repeat domains and flanking regions, binds to microtubules, and mediates tau aggregation. The projection domain projects away from the microtubules, thus mediating interaction with other bindings partners of tau. The middle region (defined as amino acids 151–243) is particularly rich in prolines and contains multiple threonine T-proline and serine-proline motifs that represent targets of proline-directed kinases. In AD, these and other motifs become hyperphosphorylated and are recognised by different tau phosphorylation-dependent antibodies. All commonly used P-tau assays in clinical practice recognise either phosphorylated T181 or T231 [80].

In humans, tau is encoded by the *MAPT* (microtubule associated protein tau) gene located on the long arm of chromosome 17 (17q21.31). The gene comprises sixteen exons [80]. Within the adult CNS, six major isoforms are expressed that are generated by alternative splicing of exon 2 (E2), E3 and E10 [81]. Each of the two exons E2 and E3 encodes one 29-residue near-amino-terminal insert (N) [80]. Accordingly, isoforms containing none, one or two of these inserts are designated 0N, 1N and 2N, respectively. Additionally, isoforms are categorised depending on whether they contain three or four carboxy-terminal repeat domains (3R or 4R, respectively). The second of the four R repeats is encoded by E10 and is not included in 3R tau. From a molecular perspective, “tauopathies” are classified into three groups based on the tau isoforms found in the aggregates: 4R tauopathies (e.g., progressive supranuclear palsy, corticobasal degeneration and argyrophilic grain disease), 3R tauopathies (e.g., Parkinson’s disease) and mixed 3R + 4R tauopathies (e.g., AD) [80]. Figure 2A provides a schematic of the *MAPT* gene and the six main splice variants of tau that are found in the human brain [80].

Tau is a phosphoprotein with 85 theoretical phosphorylation sites in the longest isoform (2N4R) [80,82]. Tau phosphorylation is developmentally regulated with fetal tau carrying an average of seven phosphates per molecule [83], while normal adult tau is phosphorylated only at two residues per molecule [80]. In AD, tau is hyperphosphorylated: an autopsy series reported approximately eight phosphates per molecule [84]. However, due to the postmortem interval during which there is marked phosphatase-mediated dephosphorylation of soluble tau, the true phosphorylation level before death is likely even higher [85]. Many phosphorylation sites cluster in the proline-rich middle region, connecting the N-terminal projection domain with the C-terminal assembly domain (Figure 2B) [80]. It contains multiple threonine-proline or serine-proline motifs that are the targets of proline-directed kinases [80]. In AD and other tauopathies, these and other motifs become hyperphosphorylated and can be recognized by several tau phosphorylation-dependent antibodies [80]. Monoclonal antibodies that are most commonly used in clinical practice to quantify CSF P-tau levels are directed against the phosphorylated threonine T181 or T231 tau epitopes [11].

Under physiological conditions, tau exerts multiple neuronal functions that differ depending on its presence in particular subcellular compartments [80]. In adult neurons, tau mainly localises to axons where it interacts with microtubules through its carboxy-terminal assembly domain [80,86,87]. Through this interaction, tau promotes the formation of microtubules and stabilises microtubule assemblies [80,88]. In addition to regulating microtubule dynamics, tau regulates axonal transport by modulating the function of the motor proteins dynein and kinesin, which are responsible for ante- and retrograde axonal transport, respectively [89]. Small amounts of tau are located within dendrites and dendritic spines, and have been proposed to modulate synaptic plasticity [90]. Additionally, tau has been shown to interact with ribosomes, thereby exerting an impact on RNA-translation [91]. Finally, tau has been detected within neuronal nuclei where it may contribute to maintaining the integrity of the genomic DNA and nuclear RNAs [92,93].

### 3.2. Tau—Its Role in the Neuropathology of AD

The identification of tau protein aggregates as the core unit of NFTs, but also the discovery of mutations in the *MAPT* gene that represent defined causes for a hereditary form of frontotemporal dementia (FTDP-17) [94,95,96], implied an important and—at least in some cases—direct role for tau in the pathogenesis of neurodegenerative diseases, even in the absence of Aβ pathology. The peculiar nature of the co-appearance of tau- and Aβ-aggregates in AD remains debated [97]. Moreover, it is not clear whether tau pathology is a downstream phenomenon of Aβ pathology in AD, or to what extent it is necessary for the occurrence of Aβ-induced toxicity [79,98]. It is believed, that tau pathology is initiated through post-translational modifications: first and foremost, phosphorylation at serine, tyrosine or threonine residues in the middle region of tau (see Figure 2B), but also acetylation, ubiquitinylation or truncation events [99]. These post-translational modifications result in the detachment of tau from microtubules, thus causing axonal microtubule disassembly, but also rendering tau more prone to form aggregates [79]. Detached tau is erroneously shifted to pre- and postsynaptic terminals where it leads to a reduction in synaptic vesicle numbers ultimately causing synaptic dysfunction and synapse loss [80,100,101,102]. Moreover, tau may form aggregates that are released into extracellular space and may be taken up by other neurons, leading to the spread of tau pathology [103,104]. Additionally, aggregated tau has lost its ability to enter the nucleus, which may result in DNA damage, due to the loss of the DNA-protective function of tau [80,105]. Finally, an intensified and aberrant tau-ribosome association has also been shown to impair RNA-translation [91].

### 3.3. From Paired Helical Filaments to the Use of Tau as Biomarker for Alzheimer’s Disease—Historical Context

Tau was first discovered in 1975 when it was found in association with tubulin purified from porcine brain tissue [106]. Subsequent research unveiled its biochemical properties, including the ability of tau to become phosphorylated, and physiological functions as a microtubule stabiliser in neurons and other cell-types [107,108]. Moreover, early studies reported that under physiological conditions, tau was found in both phosphorylated and dephosphorylated forms, and that tau dephosphorylation promotes rapid polymerisation of microtubules [109,110]. Although this implies a regulatory function of different tau phosphorylation states, the pathophysiological significance of this finding was completely unknown [111]. In 1986, the roads of tau and AD research crossed when several research groups identified tau as the major constituent of paired helical filaments (PHF) [112,113,114,115,116,117,118,119], the core fibril of NFTs in AD [120]. At the same time, human tau was cloned and the genomic locus of the *MAPT* gene was identified on the long arm chromosome 17 [121]. Subsequently, the six major tau isoforms in the brain were described [81], tau-specific antibodies were developed [122], and abnormal posttranslational modifications of tau were identified, such as certain phosphorylation events [123].

Based on the assumption that CSF analysis may mirror pathological biochemical processes in the brain, and despite the low solubility of NFTs and their constituting aggregated tau fibrils [124,125], an early case series reported the detection and elevation of pathological forms of tau in the CSF of AD patients compared to controls, thus heralding its use as CSF biomarker for the diagnosis of AD [126]. However, the monoclonal antibody used in this study was later shown to recognise an epitope that is more widespread than initially anticipated and presents on several proteins that undergo post-translational ubiquitinylation [127]. Subsequently, a small pilot study demonstrated an increase of a pathological form of tau in the CSF of AD patients [128] using the more specific Alz-50 antibody [122].

The finding that antibodies recognising pathological forms of tau are not specific for the detection of NFTs, but also stain the cytoplasm in neurons devoid of NFTs [128,129,130,131], and the fact that PHF tau could be readily extracted from AD brain tissue using detergent-free buffers, suggested that pathological phosphorylation of tau represents an early event in the cascade of PHF and NFT formation [130,131,132], and provided a rationale for its detection in the CSF early in the disease course [129]. This fuelled the development of assays for the detection of both total tau (T-tau) and phosphorylated tau (P-tau) as AD CSF biomarkers [129,133].

### 3.4. Tau as CSF Biomarker for Alzheimer’s Disease

Today, hundreds of studies have compared CSF T-tau and P-tau levels in patients with AD and healthy elderly controls or other differential diagnoses of AD [72]. As discussed above, there are various isoforms and distinct phosphorylation states of the tau protein in the CNS [134]. The most commonly used ELISA method for T-tau is based on monoclonal antibodies that detect all isoforms of tau independently of their phosphorylation state [129]. An increase in CSF T-tau in AD of approximately 200–300% has been consistently reported [11]. The CSF level of T-tau probably reflects the intensity of neuronal damage and neurodegeneration [11]. This assumption is based on findings that in acute conditions such as stroke, there is a marked transient increase in CSF T-tau that directly correlates with infarct size [135,136]. Similarly, CSF T-tau is increased following traumatic head injury [137,138]. Furthermore, among neurodegenerative diseases, the highest rise of CSF T-tau is found in Creutzfeldt-Jakob disease, which is characterised by very rapid progression of neuronal degeneration [139], whereas only moderately increased levels are found in AD with less intense degeneration [140], and normal levels in patients with depression [11,129].

A moderate to marked increase in CSF P-tau has been found in AD patients using the commonly used ELISA methods [11]. The CSF levels of P-tau are assumed not only to reflect the phosphorylation state of tau in the brain, but also the formation and load of NFTs [11]. This hypothesis is based on the previous demonstration of a positive correlation of CSF P-tau levels and neocortical tangle pathology [74,141]. Moreover, higher CSF P-tau levels are observed in patients with faster progression from mild cognitive impairment (MCI) to AD [142], and a more rapid cognitive decline [143,144]. In contrast to T-tau levels, there is no change in CSF P-tau following acute stroke [135,136]. Thus, P-tau levels seem to be more specific to AD pathology than T-tau levels.

## 4. Alzheimer’s Disease CSF Core Biomarkers in Clinical Practice

The clinical manifestation of AD is generally preceded by a silent preclinical phase, after which the first clinical symptoms appear in the prodromal phase as mild cognitive impairment, followed by overt dementia [2]. These phases are characterised by biochemical changes in the brain that are reflected by corresponding alterations in the CSF [4]. As summarised above, many studies have reported a highly significant decrease in CSF Aβ levels, and a concomitant increase in T-tau and P-tau in patients with MCI or dementia due to AD. Indeed, the constellation of low Aβ and high T-tau and P-tau levels is commonly referred to as the “Alzheimer profile” or “Alzheimer signature” [145,146]. This combinatorial use of CSF biomarkers may be used to discriminate patients with AD from nondemented age-matched people with high sensitivity and specificity [72], but also from several important differential diagnoses, such as depression [129]. Therefore, current international research criteria of AD acknowledge the use of biomarkers, including CSF biomarkers, to demonstrate evidence of AD pathology, and to discriminate between dementia or MCI due to underlying AD pathology and alternative pathologies [147,148,149].

Nonetheless, the invasive nature of lumbar puncture [150], costs, the lack of standardised analysis procedures across different laboratories [151], and the resulting differences in cut-off values [152], have precluded a more widespread use and incorporation into guidelines for daily clinical practice. Recommendations for the clinical use of the core CSF biomarkers have been formulated in a recent consensus report by members of the Alzheimer’s Biomarkers Standardization Initiative [153].

Most AD patients are diagnosed using clinical criteria based on the National Institute on Aging and the Alzheimer´s Association Workgroup guidelines [147,154]. These criteria are based on the recognition of the dementia syndrome, the classic features of AD, and the exclusion of other nondegenerative causes of dementia. However, in comparison with neuropathological diagnosis, accuracy is limited with a sensitivity of 71–87% and a specificity of 44–71% [153,155]. The proportion of misdiagnosed patients is even higher in cases of early-onset AD, cases of AD with atypical presentations, or in cases of dementia with mixed etiologies [153,156]. Therefore, it was recommended that all patients with memory complaints or related cognitive deficits should be considered for lumbar puncture and subsequent AD CSF biomarker analysis [153]. More specifically, CSF biomarker analysis should be offered to patients with early-onset dementia, MCI, or an atypical clinical presentation of a dementia syndrome with a complex differential diagnosis [153].

In each of these cases, AD CSF biomarkers are important to distinguish cognitive deficits due to AD from those stemming from non-AD conditions. The latter are a heterogeneous group of disorders, including other neurodegenerative disorders, but also psychiatric, and infectious diseases. Atypical AD presentations make up approximately 10% of AD cases and include focal variants, such as logopenic aphasia, posterior cortical atrophy, and the frontal variant of AD [157]. The correct diagnosis of AD in these cases can only be ascertained by the presence of in vivo evidence of typical AD pathology through use of appropriate biomarkers [153]. Likewise, it has been estimated from autopsy results that approximately 50% of all AD cases have additional non-AD pathology, thus presenting with mixed dementia, impeding clinical diagnosis [158]. In the same way to the ascertainment of correct diagnosis in patients with atypical AD presentation, in vivo evidence of AD pathology is indispensable for diagnosing the AD component of mixed dementia [153]. Finally, individuals in the early stages of AD pathology (i.e., preclinical and prodromal AD) are the most likely to benefit from disease-modifying therapies once they become available. Hence, it is important to be prepared when effective drugs for these stages of AD become available, and that the early identification of AD by use of AD CSF biomarkers is standardised [153]. For these reasons, international research criteria for the diagnosis of AD in its predementia phase demand the use of biomarkers [149,159,160].

In general, combined analysis of CSF biomarkers should result in a better diagnostic performance than any biomarker alone. Therefore, it was recommended that all three classical CSF AD biomarkers (Aβ, T-tau, P-tau) should be analysed for accurate diagnosis [153]. However, the analysis of all three biomarkers is not necessary in all circumstances as different sets of biomarkers predict the probability of disease depending on the clinical scenario: if the indication for CSF AD biomarker analysis is not clear, it is advised to have all three biomarkers analysed. In contrast, for the differentiation of AD from controls, a combination of Aβ and T-tau may suffice, whereas for the differentiation of AD from non-AD dementias, a combination of Aβ and P-tau yields the best results [153].

Standardisation of pre-analytical aspects of CSF AD biomarker testing should be performed according to recent consensus recommendations [151]. The cut-off values used by different laboratories to distinguish between the normal and pathological range differ widely. Even when the same assay is used, inter-laboratory variability differs by up to 30% [152]. Naturally, the setting of appropriate cut-off values is inherently imprecise and can only yield the best compromise, as it is based on samples from clinically diagnosed but usually not pathologically confirmed cases of AD, non-AD dementia, and healthy controls. Even if clinical diagnosis is correct, AD pathology may be present for decades before clinical disease onset and reflected by corresponding changes in CSF AD biomarker levels. Therefore, presumed healthy controls or non-AD dementia patients may have underlying AD pathology, making them already positive for amyloid markers [4,161]. As a result, the use of “grey zones” in which there is doubt about the result is considered good clinical practice [153]. A grey zone is typically defined as 10% of the actual cut-off value reaching from the cut-off value into the pathological range (i.e., a 10% decrease in the case of Aβ, and a 10% increase in the case of T-tau and P-tau) [153]. Therefore, to consider a value as positive, this should be at least 10% below (for Aβ) or above (for T-tau and P-tau) the actual cut-off value. This practice minimises false-positive diagnosis based on biomarkers, which may be important as long disease-modifying therapies are not available [153]. Adjustment of cut-off values for potentially confounding factors such as age or the *APOE* genotype is not required [153].

**Figure 3:**
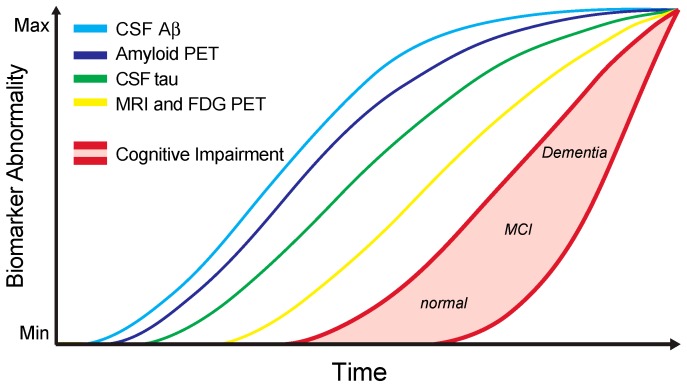
The staging of Alzheimer’s disease according to core biomarkers. The graph demonstrates the classical model for temporal changes of AD core biomarkers along the cognitive continuum from health to dementia. According to this model, a reduction of CSF Aβ levels represents the earliest detectable biomarker change mirroring AD pathology, closely followed by positron emission tomography (PET) amyloid imaging. These two markers of amyloid pathology may reach their maximum pathological values even before the onset of dementia. Biomarkers of neuronal degeneration, including CSF tau, fludeoxyglucose (FDG)-PET, and structural brain imaging, reach abnormal values at a later point compared to amyloid biomarkers. Cognitive impairment is not illustrated as a single curve, but as a zone to account for variations according to AD susceptibility due to genetic or environmental factors. The temporal evolution of AD biomarkers must be considered when interpreting test results in the work-up of patients with cognitive impairment [161].

Finally, for the interpretation of AD biomarker results and their correlation with clinical symptoms, it is important to take the long and continuous AD disease course into account that progresses from normal cognition to MCI, and finally reaching dementia [2]. This process is accompanied by distinct trajectories of different kinds of biomarkers that change over time before and throughout the disease course (Figure 3) [4,153,161]. As part of the interpretation of CSF biomarker results, integrative analysis of other biomarker modalities is important (Figure 3). Foremost, these include structural, functional, and molecular brain imaging techniques reflecting AD-related pathologies and neurodegeneration [162]. Additional biomarker modalities include peripheral blood biomarkers [163], and electrophysiological tools [164,165].

Structural magnetic resonance imaging (MRI) enables the detection of both global and regional patterns of cerebral atrophy. The major underlying neuropathology is thought to be the loss of neuronal processes and neurons [162]. Brain atrophy, as determined by MRI, initially affects structures of the medial temporal lobe, followed by the involvement of the temporal neocortex and subsequently all other neocortical association cortices. Neocortical atrophy typically evolves symmetrically [162,166]. The sequence of atrophy progression on MRI is highly similar to the spatial spreading of neurofibrillary tangles in histopathological studies [5,162,166]. In addition to structural MRI, FDG-PET represents another brain imaging modality that is routinely used in clinical practice [162]. It allows the visualisation of brain glucose metabolism, with synaptic activity as its principle underlying neurophysiological component [162]. Patients suffering from AD typically show severe hypometabolism in association and limbic cortical areas [162,167]. Both cerebral atrophy and hypometabolism start early in the disease course, even before the onset of clinical symptoms, and subsequently correlate with the clinical progression of cognitive decline (Figure 3) [4,161,162,168,169,170,171,172,173]. However, atrophy and hypometabolism are relatively nonspecific results of neuronal damage and reduced synaptic activity, and as such may be biased by the presence of other neurological diseases or other confounders of MRI brain volume changes and FDG-PET hypometabolism, respectively [162].

A third brain imaging modality with an established role in the clinical or scientific work-up of AD patients is amyloid PET [162]. Amyloid PET allows the direct visualisation of cerebral Aβ load by positron emission tomography using Aβ ligands, such as the 11c-labelled Pittsburgh Compound B (^11c^PIB). Therefore, it serves as an in vivo marker for the presence of Aβ pathology, that yields similar diagnostic information compared to CSF Aβ levels [162]. Indeed, like a decrease of CSF Aβ levels, amyloid PET abnormalities become evident early in the disease course before the onset of clinical symptoms. However, in contrast to CSF Aβ levels, maximum levels of abnormality are typically not reached as quickly, thus rendering amyloid PET more useful for the determination of progression of Aβ pathology compared to Aβ CSF levels (Figure 3) [4,161,162]. Nonetheless, amyloid PET does not reach the performance of structural MRI and FDG-PET as surrogate marker of disease progression (Figure 3).

## 5. Additional Candidate Biomarkers

The pathophysiology of AD is complex and not limited to amyloid plaques and neurofibrillary tangles, but also includes responses of the innate and adaptive immune system, gliosis, loss of synapses and neurons [6]. All these neuropathological characteristics are typically observed in AD brain tissue and mirrored by alterations in the composition of brain-derived molecules in the CSF. Consequently, in addition to the three core AD CSF biomarkers (Aβ, T-tau, P-tau), many other molecules have been proposed or investigated as candidate biomarkers for differentiating AD from healthy controls or other causes of dementia, for monitoring disease progression or target engagement of novel candidate drugs, or for predicting the rate of cognitive decline [174]. From a pathophysiological perspective, these candidate molecules can be classified as markers of APP processing, synapse loss and neurodegeneration, neuroinflammation and astroglial responses, or oxidative stress [174].

Importantly, for the differential diagnosis of AD it is unlikely that any of the hundreds of proposed AD CSF biomarker candidates will reach the diagnostic performance of the three core biomarkers Aβ, T-tau and P-tau, since these reflect the pathological hallmarks of the disease and have been verified in large prospective studies and meta-analyses [72]. However, new biomarkers will equip the toolbox with a set of markers that collectively cover the entire spectrum of molecular events in AD. Usage of such panels in longitudinal clinical studies could increase our knowledge of the temporal evolution of molecular pathologies during the progression of AD. Moreover, even single molecular biomarkers may become powerful tools for the evaluation of the therapeutic efficacy of novel drug candidates. It is likely that therapeutic strategies that do not target Aβ- or tau-pathology related mechanisms, also require biomarkers that are unrelated to Aβ and tau to assess the desired target engagement. Regardless of whether certain pathological processes are disease-specific or not, they may represent drugable targets. Indeed, in the absence of curative or disease-modifying treatments for AD, targeting disease-associated processes such as inflammatory responses or oxidative stress may represent a potential therapeutic approach that could mitigate the clinical course of this devastating disease [175]. Therefore, identifying and monitoring the presence of drugable, disease-associated processes through the use of appropriate biomarkers will facilitate future clinical treatment studies.
